# Membrane Separations in Biomass Processing

**DOI:** 10.1002/cplu.202400497

**Published:** 2024-11-21

**Authors:** Anurag S. Mandalika, Troy M. Runge, Arthur J. Ragauskas

**Affiliations:** ^1^ Assistant Research Professor, Center for Energy Studies Louisiana State University 93 S Quad Dr, 1115 Baton Rouge LA 70803; ^2^ Professor of Biological Systems Engineering and CALS Associate Dean for Research, 2121 Wisconsin Energy Institute Building University of Wisconsin-Madison 1552 University Ave Madison WI 53726; ^3^ Governor's Chair for Biorefining, Joint Institute for Biological Sciences, Biosciences Division Oak Ridge National Laboratory 1 Bethel Valley Road Oak Ridge TN 37831

**Keywords:** Biomass, Separations, Membranes, Biofuels, Bioprocessing

## Abstract

The development of integrated biorefineries and the greater utilization of biomass resources to reduce dependence on fossil fuel‐derived products require research emphasis not just on conversion strategies but also on improving separations associated with biorefining. A significant roadblock towards developing biorefineries is the lack of effective separation techniques evidenced by the relative deficiency of literature in this area. Additionally, high conversion yields may only be realized if effective separations generate feedstock of sufficient purity – this makes research into biomass conversion strategies all the more critical. In this review, the challenges associated with biomass separations are discussed, followed by an overview of the most appropriate separation strategies for processing biomass. One of the unit operations most appealing for biorefining, membrane separations (MS), is then considered along with a review of the recent literature utilizing this technique in biomass processing.

## Graphical abstract

Separations are critically important in enabling biorefineries of the future. We review the background and current applications of membranes in biomass refining, challenges associated with their implementation, and future prospects for this technology.

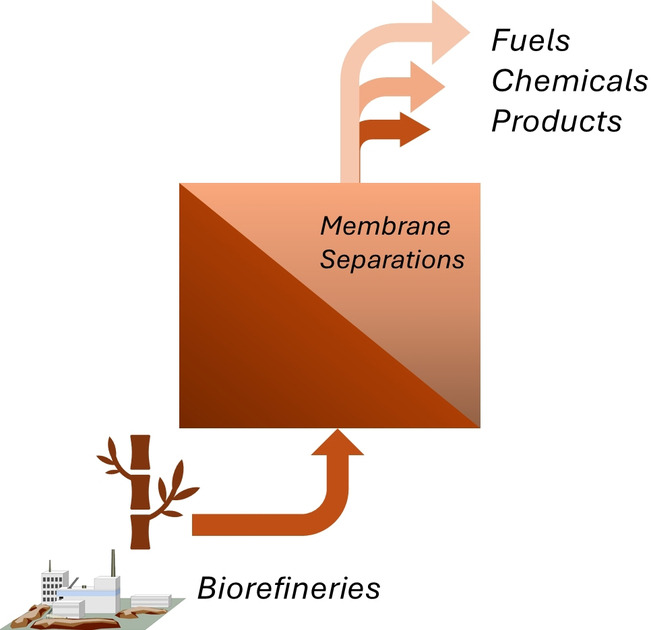

## 1 Background

The utilization of biomass feedstock, residues, and waste streams from bioprocessing operations is severely hampered by the inability to efficiently and effectively separate target streams from each other. This leads to insufficient utilization and represents a significant bottleneck in the *valorization* (providing additional value) of biomass resources for the development of integrated biorefineries; the latter represents a building block in the transition from fossil fuel to bio‐based societies. Although utilization approaches for several biomass‐derived products and constituents have been thoroughly explored, their upstream separation approaches have been sparsely studied, and this represents an engineering opportunity that needs to be addressed.

**Figure 1:**
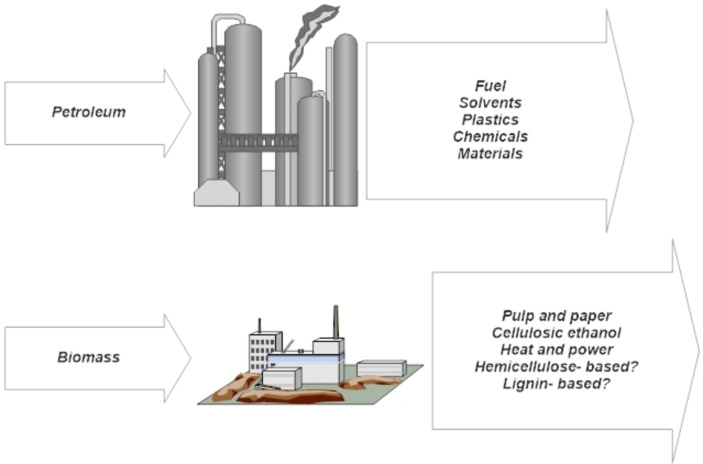
The integrated biomass refinery is imagined as an analog of the petroleum refinery‐ an appealing but limited and simplistic concept.

Integrated biorefineries, the stepping stone towards realizing societies that are less reliant on fossil fuel sources for their energy and materials needs, are defined as an ′overall concept of a processing plant in which biomass feedstocks are converted and extracted into a spectrum of valuable products.[^1^, ^2^, ^3^] The concept of the integrated biorefinery has been reimagined as an analog (for the replacement) of the ubiquitous petroleum refinery,[^3^, ^4^] where a single feedstock, crude oil, is delivered into a myriad of valuable products (fuel, solvents, plastics, resins, adhesives, etc.) via the process of fractional distillation, by which individual products are neatly separated according to their boiling points. However, the analogy is incomplete as not only are there a variety of physical, chemical, and biological transformations that are possible, but biomass does not afford a neat separation strategy by exploiting a single physical or chemical characteristic of the feedstock (Figure 1). Clark^[5]^ has offered some much‐needed perspective on the comparisons between petroleum refineries and biorefineries, calling the analogy ′superficial′. To supplement this claim, distinctions are made between the complexity of cracking biomass in comparison to petroleum and separation difficulties encountered when dealing with biomass products. For example, in fermentation processes where the products stream is very dilute (12–14 % in concentration), highly polar, and oxygenated, separation challenges are estimated to be an order of magnitude greater than those encountered in petroleum processing.

Over the last couple of decades, some highly cited reviews in this area,[^6^, ^7^] have popularized this analogy—although these studies have been instrumental in generating the momentum for research and development, the emphasis has been almost heavy‐handedly focused on novel conversion approaches and not enough on devising engineering solutions that address separation challenges. There are fewer studies and reviews^[8]^ on biomass separations, which go hand in hand with conversion and upgrading. Other authors[^4^, ^9^] have made similar arguments, noting the lack of emphasis on separation techniques, among other aspects of bioprocessing. Ragauskas, et al.^[10]^ acknowledge the importance of separations in their review outlining the path forward for biorefineries—separations can account for up to 80 % of the process costs of mature chemical processes, but even this narrative has not necessarily been the norm. After all, high conversion efficiencies based on targeted reaction schemes are only possible if adequate separation is achieved in the first place. Indeed, Seader and Henley^[11]^ designate chemical reactions and separation of the chemical mixtures as critical operations in any chemical process. The inherent physical and chemical complexity of biomass feedstocks necessarily requires adopting several targeted strategies, and the research into *biomass separations* has lagged that of *biomass utilization*.

Following effective separation, lignocellulosic components and streams need to be concentrated to ready them for valorization. Concentrated streams are beneficial for process economics and remain an important consideration for many unit operations in bioprocessing. Product streams are easier to handle and store when they are not dilute, representing an added benefit. In fact, concentration can be thought of as a subset of separation, in which the separation is from the solvent and any other impurities.

In this review, following a brief introduction to the physicochemical structure of lignocellulosic biomass, the challenges associated with biomass separation, primarily arising due to its heterogeneity, are outlined along with a discussion of potential separation unit operations. Recommendations are made to choose the most appropriate separation techniques for the biomass pretreatment process and biorefining, emphasizing potential applications of membrane separations (MS).

## 2 Overview of Lignocellulosic Biomass

It is estimated that global annual terrestrial biomass production accounts for ~100 billion tons, with another ~50 billion tons produced aquatically.^[12]^ In the United States, the recently updated Billion‐Ton 2023 (BT23) study estimates that biomass production potential in a mature market in the US can range between 1.1 and 1.5 billion tons annually, which would be equivalent to producing over 60 billion gallons of renewable carbon‐based liquid fuels.^[13]^ This sizeable availability, coupled with the renewable nature of the resource, makes biomass resources an ideal candidate for consideration towards biorefining and biofuels applications.

Lignocellulosic biomass is broadly comprised of cellulose, hemicelluloses, lignin, extractives, tannins and other polyphenols.^[14]^ All constituents play vital roles in the growth and development of the plant. Cellulose is a structural polymer accounting for the majority of the lignocellulosic plant material (40–45 % by dry weight in the secondary cell walls), hemicelluloses aid in structure and promotes cross‐linking of cellulose microfibrils, lignin acts as the hydrophobic matrix to hold the cell wall together and repel water (~15–36 % of the dry weight of wood), extractives and plant polyphenols are present in small amounts, and primarily have roles in plant defense against microbial pests.

Cellulose is a homopolysaccharide composed of β‐d‐glucopyranose units bonded by (1–4)‐glycosidic bonds. It is a polymer composed of cellobiose units (a molecule formed by the condensation of two glucose units). The chemical formula of cellulose is (C_6_H_10_O_5_)_n_, where *n* is the number of repeating units of glucose, also referred to as the *degree of polymerization* (DP), which varies by individual feedstock and can range up to 8,000. The cellulose structure is a highly linear molecule. The polymeric linkages form the chains in an extended manner, enabling these to fit together tightly over long segments, thus giving rise to powerful associative forces that contribute to the strength of cellulosic materials. Individual cellulose molecules undergo self‐assembly, aided by associated hemicelluloses, and anywhere between 18 and 24 of these linear chains pack into structures called protofibrils. Packing of these protofibrils gives rise to microfibrils, which further aggregate to give rise to the familiar cellulose fibers.^[15]^ Inter‐ and intramolecular hydrogen bonds stiffen the cellulose molecules, and cellulose sheets are held in place by weak inter‐sheet van der Waals forces.^[16]^

Hemicelluloses are the second‐most abundant polysaccharide class in nature.^[17]^ Unlike cellulose, hemicelluloses are not homopolysaccharides, being composed of various pentose and hexose sugars and uronic acids. For this reason, the hemicellulosic sugars are also referred to as polyoses. Individual sugar monomers found in plant hemicelluloses are xylose and arabinose (pentoses) and glucose, galactose, and mannose (hexoses). The main chain of the polyoses can contain one carbohydrate (homopolymer) such as xylan or two or more carbohydratess (heteropolymer) such as glucomannan. The principal pentose sugar is β‐d‐xylopyranose, which has the 2 C and 3 C carbons available for O‐linked substitution as in xylan and is used as the most straightforward representation for hemicellulose. Hemicelluloses are usually bound to cellulose via hydrogen bonds, which helps stabilize the cell wall matrix and renders the cell wall insoluble in water. Compared to cellulose which has crystalline regions, hemicelluloses are amorphous and much more susceptible to attack by acids, rendering hydrolysis with dilute acid a much easier process. Xylans are the most abundant hemicelluloses, these being heteropolysaccharides with homopolymeric backbone chains of 1,4‐linked β‐d‐xylopyranose units. Besides xylose, xylans may also contain arabinose, glucuronic acid, or its 4‐O‐methyl ether, and may be acylated with acetate, ferulate, and/or *p*‐coumarate.^[17]^

Lignin is a polymer of aromatic subunits derived from phenylalanine. It functions to provide additional rigidity and compressive strength by forming a matrix around the holocellulosic polysaccharides and renders the cell walls hydrophobic and impermeable to water. It has been estimated that 15–36 % of the mass of dry wood is lignin, making it one of the world's most abundant polymers.^[18]^ Lignin is a complex polymer formed by the combinatorial oxidative radical coupling of 4‐hydroxycinnamyl alcohols (monolignols), although there are many examples of other phenolics being incorporated into the structure of lignin, besides monolignols.^[19]^ The coupling occurs after the enzymatic dehydrogenation of phenylpropanoids, leading to the formation of the lignin polymer. Most plant lignin is derived from three units: guaiacyl (G), syringyl (S), and *p*‐hydroxyphenyl (H) moieties, their relative amounts differing among biomass types. Softwood lignin is primarily composed of guaiacyl units, which originate from the predominant precursor, *trans*‐coniferyl alcohol. Both syringyl and guaiacyl units best represent hardwood lignin, originating from *trans*‐sinapyl and *trans*‐coniferyl alcohols, respectively.

In addition to the macromolecular compounds (holocellulose and lignin) that constitute woody cell wall, biomass also contains minor components that are soluble in water or organic solvents, which are referred to as extractives and are not part of the cell wall. These are generally distributed in the lumen and specific tissues, such as the resin canal, and do not interact with the cell wall components of wood. These are primarily low molecular weight compounds and typically make up less than 5 % of the wood in relation to cellulose, hemicellulose, and lignin.^[20]^ The types of extractives can be broadly classified into three categories:

- Lipid extractives, comprising of aliphatics (fatty acids, fatty acid esters, waxes, and suberin (a polyester), and terpenoids (monoterpenes, diterpenes, and triterpenes).
- Phenolic extractives comprise simple phenolics (such as gallic acid and vanillin), stilbenes, flavonoids (most abundant phenolic extractives), and lignans.
- Other extractives (alkanes, proteins, monosaccharides, and various derivatives thereof).

Terpenoids are generally limited to softwood species only, occurring as constituents of essential oils. Terpenes are generally hydrocarbons, whereas terpenoids are compounds that contain oxygen atoms with functional groups such as alcohols, aldehydes, and ketones.^[21]^

Polyphenols (of which tannins are a subcategory) originate along with lignin from the phenylpropanoid and shikimate pathways, with the flavonoids arising from a p‐coumaryl‐CoA precursor via the action of chalcone synthase. Another route leading from p‐coumaryl‐CoA is due to the action of hydroxycinnamyl‐CoA shikimate/quinate hydroxycinnamyltransferase (HCT), which leads to the formation of the monolignols for guaiacyl (G) and syringyl (S) lignin.^[22]^ Tannins, a subset of extractives, constitute a class of plant polyphenolics that are water‐soluble and are identified by their ability to precipitate proteins, a property referred to as astringency—exploited in applications such as tanning.^[23]^ Vegetable tannins are widely referred to as plant polyphenols, where the latter name refers to compounds with more than one arene ring.^[24]^ Having undergone several attempts at definition, one of the most specific descriptors for plant polyphenols comes from Quideau, et al.^[25]^ who define these as plant secondary metabolites that are derived exclusively from the shikimate‐originating phenylpropanoid and/or the polyketide pathways, with compounds containing at least one phenolic group while lacking any nitrogenous moieties.^[24]^ The ensuing discussion will treat these two classes as the same, and both terms may be used interchangeably. Tannins can belong to one of three categories: hydrolysable tannins (such as gallo‐ and ellagitannins), condensed tannins (proanthocyanidins), and phlorotannins, such as those found in red‐brown algae.^[25]^

The guiding criteria for the classification of tannins rely on strict structural requirements (water‐soluble plant phenolics with molecular weights ranging from 0.5 to 4 kDa, and the presence of 12 to 16 phenolic hydroxyl groups on 5 to 7 aromatic rings for each 1 kDa of molecular mass), and the ability to form complexes with biomolecules,^[25]^ a characteristic property of tannins that enabled their widespread historical use in the leather industry. In plants, tannins can accumulate in large quantities (exceeding 10 % of the dry mass) in tissues such as bark, wood, leaves, fruits, and roots.^[23]^

The final main constituent of dry biomass feedstock which has an impact on its application is the mineral ash content. This is primarily taken up by plant tissues from the soil and the ash composition is regiospecific to where the plant grows. Depending on the type of biomass feedstock, the inorganic matter can range anywhere between 0.1 and 46 % (with an average of 7 % on a dry basis).^[26]^ The mineral content of biomass is not typically considered a process stream for valorization, although there have been research applications for use of ash in cements, concrete, and adsorbent media. Historically, the mineral content of biomass feedstocks has been viewed as a deterrent in its valorization with pretreatment techniques employed to lower the biomass ash content in certain feedstocks.

### 2.1 Biomass Waste Streams and Availability

Most biorefining operations typically focus on one product of value, thereby relying on the extraction of mostly one biomass substrate via fractionation and conversion to derive products such as cellulose pulp, or ethanol (from cellulose). The production of furfural (and other furan‐based chemicals) relies on the extraction of pentose (5 C) carbohydrates, which are derived from hemicelluloses.[^27^, ^28^] A large amount of lignin is generated annually from the pulp and paper industry, amounting to over 300 million tons per year. Of this, just 50 million tons are extracted, and only 2 % of that, or 1 million tons, is actually commercialized, mostly in the form of lignosulfonates.^[29]^ The majority of industrial pulping processes adopt the *kraft* alkaline process, in which condensed lignin is generated and is typically burned to generate energy for the plant operations and recover the inorganic chemicals used in the process. There are fewer, if any, current commercial examples of all three primary fractions that are extracted and separated for targeted conversions. The main exception to this are thermal conversions (based on combustion, gasification or pyrolysis) where biomass is generally deconstructed as a whole to produce fuel precursors.

Globally, lignocellulosic biorefineries have been prioritized in several countries, including the US, European nations, the UK, Brazil, China, India, Thailand, Australia, New Zealand, Bolivia, Colombia, South Africa, South Korea, etc., all of which have roadmaps and policies that promote biofuels and biorefineries.^[30]^ According to the US Department of Energy (DOE), there are currently 51 active biorefineries in the US that are co‐funded by the agency's Bioenergy Technologies Office (BETO).^[31]^ These projects range from technology readiness level (TRL) ranging between the modeling and analysis stage to TRL 3 (bench scale) to TRL 8 (commercial scale). Recently, BETO established the Bioprocessing Separations Consortium led by several US national laboratories, with the goal of scaling up cost‐effective separation technologies, particularly in the area of biofuels production.^[32]^ These federal investments highlight collective efforts in the area of biorefining and the relative importance placed on separation technologies which are critical for biorefinery feasibility.

## 3 Separation Strategies for Lignocellulosic Biomass Constituents

As previously discussed, biomass refineries are conceptualized alongside the model of petroleum refineries, and while the analogy of going from a single feedstock to a wide variety of products is appealing, this picture only tells half the story—separation of biomass products is a very different challenge. Not only are most biomass‐derived products non‐volatile (in comparison to petroleum‐derived products), ruling out the broad application of distillation as a unit operation, biomass process streams are often very dilute, requiring the removal of large amounts of water. Separation operations in chemical processes (especially water) require significant energy inputs and are estimated to account for anywhere between 60 and 80 % of the total costs.^[33]^ In comparison, separations in oil refining can amount to between 40 and 50 % of total costs.^[4]^

Separation strategies in biorefineries can be summarized into a few categories:^[4]^ phase change (e. g., distillation, precipitation, etc.), affinity‐based (e. g., liquid‐liquid extraction, absorption and adsorption, etc.), size and charge‐based (ion‐exchange, membrane separations, etc.), and reactive separations (reactive distillation, extractive fermentation, etc.). Table 1 reviews some relevant studies that encompass these categories.

**Table 1:** Examples of some separation strategies employed in biorefining.

Separation	Process	Product(s)	Applications References
*Phase Change*			
	Pyrolysis	Benzene, toluene, and xylene (BTX)	Flash pyrolysis of BTX from continuous flash distillation of bio‐oils^[53]^
Distillation	Fermentation	Ethanol	1. Salt extractive distillation with electrodialysis and spray‐drying for salt recovery^[54]^
			2. Extractive distillation: extractive dividing wall column (EDWC) using glycerol as a solvent^[55]^
			3. In situ vacuum distillation of ethanol from stillage to reduce toxicity and recycle cellulose^[56]^
		Lactic Acid	
Membrane Distillation		Ethanol	1. Fermentation with membrane distillation[^57^, ^58^]
			2. Coupling distillation with vapor permeation to dehydrate ethanol^[59]^
Double Effect		Ethanol	Reduction in steam consumption using double effect distillation[^60^, ^61^]
Affinity‐Based			
Liquid‐liquid Extraction	Pyrolysis	Phenols	Extraction in dichloromethane^[62]^
			ILs and DES^[63]^
Salting Out	Fermentation	Isobutanol	Salting out using potassium phosphate to increase purity of isobutanol^[64]^
Adsorption	Fermentation	Itaconic acid	Separation of itaconic acid from model solutions in glucose, and from fermentation broth by adsorption onto cross‐linked polymers^[44]^
		Acetic acid, formic acid	Adsorption of acetic and formic acids from aqueous solutions onto hydrotalcites^[45]^
*Charge‐Based*			
Ion‐Exchange	Fermentation	Inhibitors	
	Lignocellulosic processing	Salts	Electrodialysis for wastewater effluents from biorefining^[65]^
	Fermentation	Lactic acid	Purification of lactic acid from dried distillers’ grains solids (DDGS) hydrolysates^[66]^
	Fermentation	Lactic acid	Recovery from mixed food waste fermentation broth^[67]^
Reactive			
Reactive Distillation	Fermentation	Lactic acid	Reactive distillation within a PI framework^[68]^
	Dehydration	Furfural	Increase in furfural yield by reactive distillation and separation of furfural^[27]^
	Polymerization	Polyesters	Polyesterification to produce biomass‐derived polyesters using reactive distillation with a divided wall column^[69]^
	Transesterification	Biodiesel	
Extractive Fermentation	Fermentation	Butanol	Extractive fermentation to produce acetone‐butanol‐ethanol from hydrolyzed cocoa pod husks using immobilized cells^[70]^
Reactive MS	Gasification	Hydrogen	Separation of hydrogen from syngas using carbon molecular sieves^[71]^

While distillation is the predominant separation strategy employed in chemical processing,^[34]^ its applications in biomass processing are limited by the temperature sensitiveness of biomass constituents. The high reactivity of biomass compounds with increasing temperatures affects the product stream. Additionally, distillation is an energy‐intensive operation[^35^, ^36^] which can increase production costs, hence, the need to find alternatives to this unit operation where possible.

### 3.1 Affinity‐Based

Solvent extraction (or liquid‐liquid separations) finds applications in the chemical and bioproducts industries, alongside the food, polymer, and pharmaceutical industries. The advantages of this unit operation are cited to be mild operational conditions and the ease of process control.^[37]^ Ionic liquids (IL) in general, and deep eutectic solvents (DES) in particular, find applications as solvents in biorefining operations.[^38^, ^39^, ^40^, ^41^] Although the selectivity offered by ILs is high, their commercial implementation is hindered by high costs, concerns of toxicity, and non‐biodegradability.^[42]^ In a different vein, the scalability of DES is challenged by effective recovery and recycling, which remains an active area of investigation.^[43]^ Fewer studies investigate the applications of adsorption‐based[^44^, ^45^] (surface) and absorption‐based separations (bulk) in biorefining. There is far more literature pertaining to these two separation strategies in the context of biomass‐based sorbent materials, though this is beyond the scope of this review.

### 3.2 Charge‐Based

Charge‐based separations include ion exchange, which is a standard, proven technology in the chemical industry. Ion exchange also has potential applications in biorefineries, particularly for the recovery of chemicals such as lactic acid.^[46]^ Challenges for the scaleup in extractive fermentation using ion‐exchange resins include the production of large amounts of liquid waste streams during elution, chemical stability and longevity of membrane materials,^[47]^ etc.

### 3.3 Reactive

Reactive separation processes combine chemical reaction(s) and physical separation(s) into a single unit operation with the potential for reduced capital costs, improved selectivity and conversion.[^48^, ^49^] Falling under the purview of process intensification (PI), reactive separations can allow for greater scalability and safety by reducing energy consumption, equipment volumes, and waste generation.^[50]^ Simulation studies on the use of reactive distillation techniques with heterogeneous catalysis for biodiesel production indicate lower energy requirements^[51]^ and greater profitability.^[52]^

### 3.4 Size‐Based

MS, and the myriad subcategories that comprise it, are the primary size‐based separation strategies which are discussed in greater detail in the next section.

At low concentrations of product streams that need to be purified and concentrated, water evaporation is no longer a feasible option as it becomes costly. It is estimated that evaporation requires the expenditure of 30–40 kWh/m^3^ of water, whereas membrane‐based processes such as ultrafiltration and reverse osmosis would require <5–10 kWh/m^3^.^[33]^ Studies have shown similar translation into a reduction of costs using nanofiltration over evaporation in bioprocessing operations.^[72]^

It is argued that transitioning to biorefineries from petrochemical refineries will require advanced separations such as membranes and adsorption, which has thermodynamic advantages in comparison to traditional separations such as distillation and absorption.^[73]^ MS have been advocated for their lower cost and energy requirements, ease of operation, the ability to incorporate mild processing conditions, etc..[^5^, ^33^] He, et al.^[74]^ argue that MS offer superior fractionation capabilities with reduced chemical and energy consumption, and review a broad array of applications ranging from the removal of fermentation inhibitors to algal harvesting. It is vital at this stage to provide a framework for MS, their classification, applications in bioprocessing, and challenges.

## 4 Introduction to MS

**Figure 2:**
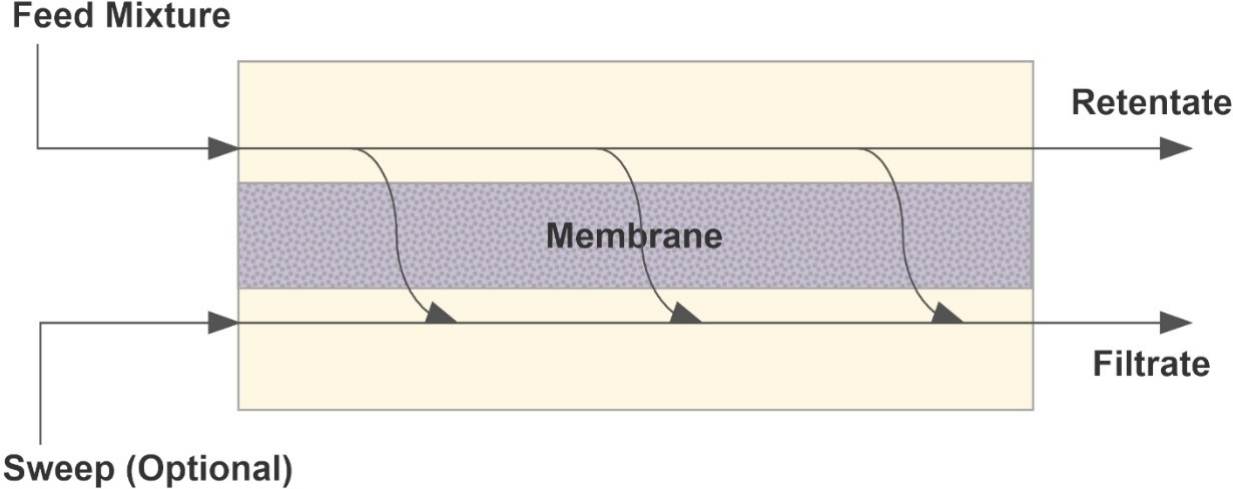
Schematic showing a generalized membrane process (adapted from Seader and Henley^[11]^).

The operation of any membrane process can be visualized using a generalized schematic, as shown in Figure 2. The ‘feed mixture’ containing the constituents that need to be separated from each other enters the membrane module as shown. Based on the characteristics of the membrane and the feed constituents, the entities that do not pass through the membrane are retained (in the stream ‘retentate’), and those that do pass through the semi‐permeable barrier are collected in the ‘filtrate’ (also referred to as the permeate). A sweep fluid is sometimes employed to remove the filtrate if necessary.^[11]^ Various modifications of this generalized approach are possible and practiced, depending on the feed mixture, processing conditions, and the desired separation outcomes, and some of these will be discussed in this section.

**Figure 3:**
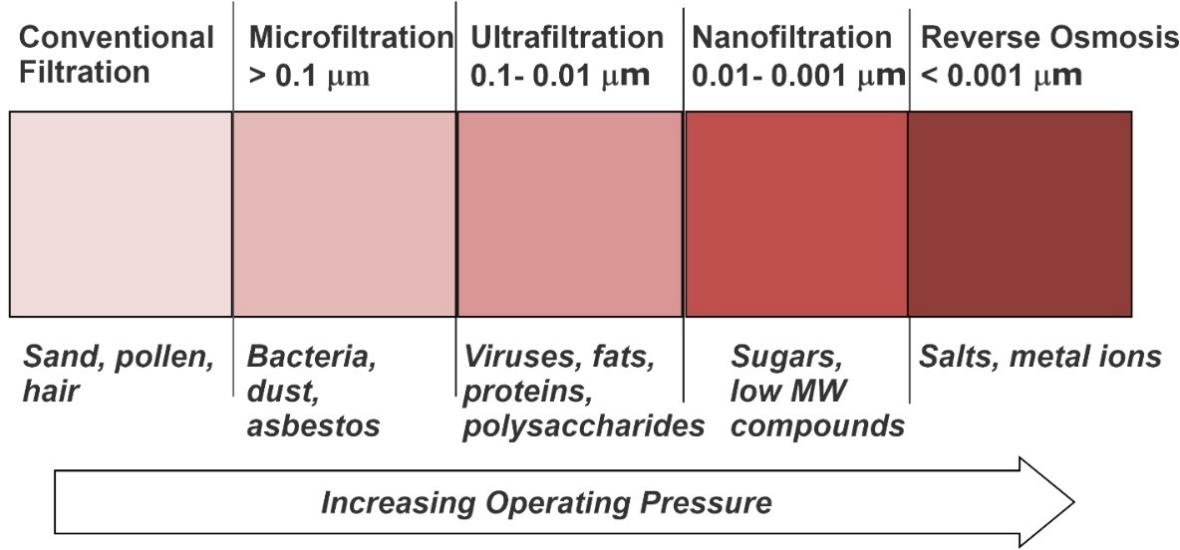
Schematic showing the subcategories and operating ranges (including pertinent classes of particles) for membrane filtration (adapted from Lee et al.^[77]^); operating pressures increase as the particle sizes become smaller, going from filtration using gravity with conventional approaches to high pressure in RO systems.

MS encompass several subtypes depending on the pore size of the membranes used, which in turn, defines their applicability for filtering certain types of constituents (Figure 3). The major subcategories are microfiltration, MF (for particles over 0.1 μm); ultrafiltration, UF (particle sizes between 0.1 and 0.01 μm); nanofiltration, NF (particle sizes between 0.01 and 0.001 μm); and reverse osmosis, RO, for even smaller particle sizes. Classes of particles that can be separated range from hair and sand that are visible to the naked eye, all the way to salts and metallic ions in water, which are an order of magnitude separated from the angstrom scale.

According to the pore size of membranes, this classification may be reinterpreted as being microporous, mesoporous, and macroporous for porous membranes. Microporous membranes contain pore sizes between 0.5 and 2 nm in diameter^[75]^ and macroporous membranes >50 nm; the middle ground is occupied by mesoporous membranes having pore size diameters between 2 and 50 nm.^[76]^

**Figure 4:**
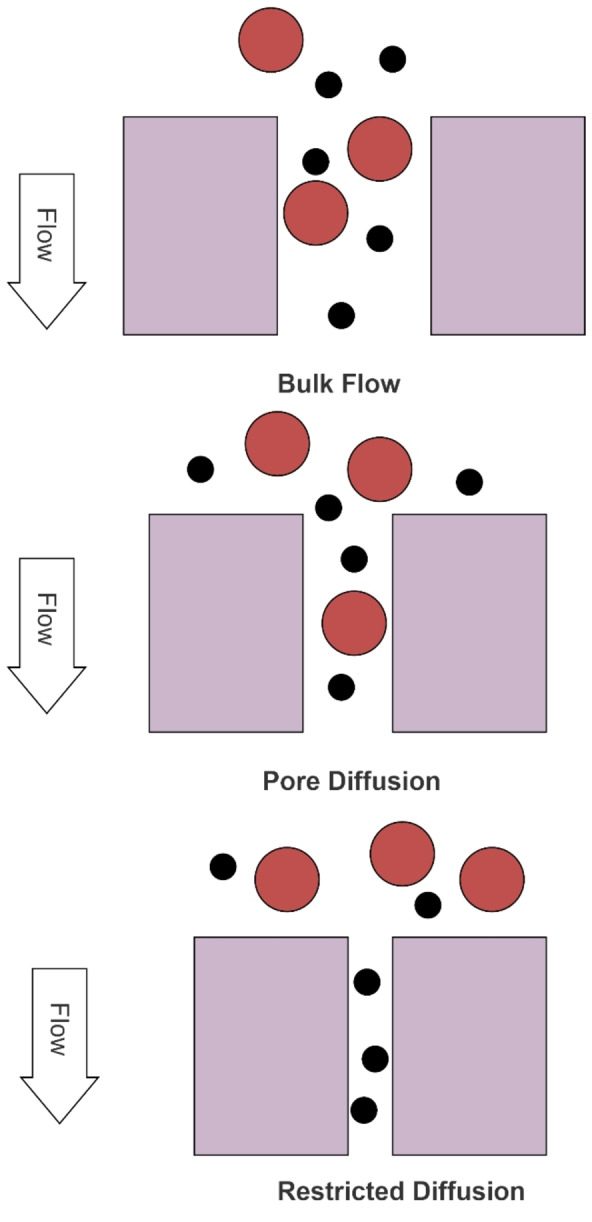
Various types of transport in porous membranes, showing diffusion of solute particles through a membrane pore as the pore size becomes comparable to the molecular size/diameter of the solute(s) present in the feed (adapted from Seader and Henley).^[11]^

Although the schematic in Figure 4 (and others such as this used in the literature) might indicate that pore size ranges and applications are strictly defined and confined, this is not actually the case; the size distinctions are somewhat broad filtration regions which can be blurred. Still, it offers a good starting point for differentiation and decision‐making based on the application of membrane filtration, as necessary. As illustrated above, UF as a unit operation is most pertinent to particle sizes between 0.1 and 0.01 μm, being most relevant to the filtration of macromolecules, which, as identified previously, represent the most under‐utilized fractions of lignocellulosic biomass‐hemicelluloses and lignin.

Membranes may be further classified based on the material of construction or their structure. Classification by structure is determined by whether the membrane is an isotropic or anisotropic structure, which indicates whether the structure is homogeneous throughout the membrane material or not. Isotropic membranes are constructed of a single material and comprise macroporous, non‐porous dense film, and electrically charged membranes, whereas anisotropic includes phase‐separation and composite membranes. Macroporous membranes, also referred to as sieving membranes, operate by size‐ exclusion, where the pore size (0.1–5 μm) of the membrane determines whether the solute(s) are filtered or retained.^[77]^ Dense film membranes typically operate based on the existence of a pressure, concentration, or an electrical differential across the ends of the membrane, which allows for diffusion of solute(s) based on the applied gradient. Electrically‐ charged membranes are characterized by the presence of charged surfaces which enhances sorption of ionic solute(s) and their retention.^[78]^

When classified according to the material of construction, membranes can be polymeric or inorganic. Polymeric, or organic, membranes are constructed from natural polymers (cellulose, rubber, etc.), whereas synthetic polymers are produced by condensation, addition reactions, or copolymerization of different monomers. Therefore, these resulting polymers offer increased flexibility in the ability to fashion linear‐chain or branched or highly cross‐linked three‐dimensional structures depending on the precursors and the polymerization strategies employed. Polymeric membranes allow for operation at temperatures below 200 °C and relatively inert chemical mixtures. When operating outside of these environments is desired, especially with corrosive environments and solvents, inorganic membranes may be used. These can be categorized into microporous ceramics, metallic, and carbon membranes, which afford greater operational flexibility.^[11]^

Polymeric membranes can be further classified based on the type of modules used: flat asymmetric (also, thin composite sheet), tubular, hollow‐fiber, and monolithic. Seader and Henley provide a good introduction to these types of membrane modules along with a compilation of pertinent characteristics such as costs and fouling resistance that in turn dictate their application for UF, MF, RO, etc..^[11]^ In general, spiral‐wound and hollow‐fiber membranes are compared to the others, with the former offering better resistance to fouling, whereas the latter allows for greater packing densities.

Inorganic membranes are sub‐divided into microporous membranes and non‐porous (or dense) membranes. As the name suggests, porous inorganic membranes contain a metallic or an inorganic support with an additional layer on top that has a different structure and morphology than the support. Materials for the construction of porous membranes include alumina, silica, zirconia, carbon, etc. In contrast, dense/non‐porous membranes are typified either by solid layers of metals or solid electrolytes and can also consist of support layers of immobilized liquid (molten salts inside ceramic supports, for example) that create a semi‐permeable layer. Materials that are used in the construction of dense membranes include palladium and its alloys, stabilized zirconia, silver, etc. Kayvani Fard, et al.^[78]^ provide a good review of inorganic membranes, particularly from the perspective of water treatment and desalination.

### 4.1 Operating Parameters and Performance Metrics

Some of the important operating considerations for MS operations are the membrane molecular weight cutoff (MWCO, Da), transmembrane pressure (TMP, psig) of the filtration, rejection coefficient (R, %), filtrate flux (L/m^2^h), etc. These terms are discussed in this section, and guidelines presented in the literature are reported.

The MWCO (in Da) is an intrinsic property of filtration membranes where filtration requirements are determined by the particle size (molecular mass) of the solute(s) that are to be retained. It is defined as the minimum molecular weight of a solute that is 90 % retained by the membrane.[^79^, ^80^] This definition is based on the (imprecise) assumption that macromolecules are spherical and all solutes are treated as structurally identical. This means that a linear molecule (such as polyacrylic acid) of a particular molecular mass that could pass through the membrane's pores is not retained whereas a globular protein of the same molecular mass is retained.^[81]^ So, although the MWCO does offer a practical approach towards size selection and retention, its utility is limited when the shapes of the macromolecules are not exactly spherical.

An alternative basis for membrane selection is based on the pore size of the membranes, reflecting the particle diameter of the solute(s) that will be rejected if they exceed this property. The pore size of a membrane is not a singular quantity, but in fact a distribution which depends on the manufacturing process, leading to potentially variable filtration outcomes for the same pore sized membrane produced by different vendors.^[82]^

One of the most important performance metrics associated with the effectiveness of a membrane separation process is the rate of rejection (defined as the rejection coefficient[^83^, ^84^]). This is a straightforward and useful measure of the membrane filtration efficiency, described using Equation 1:

(1) $$R\left(\%\right)=\left(1-\frac{C_{Fi}}{C_{F}}\right)\times 100$$

in which R is the rejection coefficient (in %), C_Fi_ and C_F_ are the filtrate and feed concentrations, respectively. When concentration polarization (formation of concentration gradients at the membrane surface different from those in the bulk solution due to selective permeability of the membrane, resulting in diminished membrane performance) does not occur in the membrane, R may be reinterpreted as the intrinsic rejection coefficient of the membrane. R is also predicted to decrease as the solute concentration increases (for pores and solutes of like charge).^[84]^

The filtrate flux, indicative of the capacity of the membrane to perform separations at scale, describes the flow rate (in L) per unit time (in h) per unit area of the membrane surface (in m^2^). Greater fluxes allow for more efficient and economically viable operations in MS systems. Monitoring the filtration flux allows for the monitoring of resistance buildup to filtration and potential fouling‐ indicating the need for cleaning and maintenance in the form of backwashing.

Transmembrane pressure (TMP) describes the pressure difference between the average of the pressures at the inlet and the outlet of the feed stream and the pressure of the filtrate stream^[85]^‐as described previously, operating pressures increase as the membrane pore sizes become smaller. It is hypothesized that greater pressures compact the membranes, thereby making the pores finer, leading to greater rejection. It is not yet known if this effect is evident in ceramic membranes. Additionally, Persson, et al.^[86]^ contend that the operating pressure has an inverse effect on the filtrate flux of the membrane, as compaction of the membranes leads to lower flow rates.

### 4.2 Transport in Membranes

When measuring the permeation effectiveness of a membrane‐solvent system, it is important to consider the transport of the solute(s) and the solvent across the membrane and shed light into the interactions of the fluid and membrane surface layers. Membranes may be classified as porous and non‐ porous (dense) membranes, based on the pore size distribution, and the former are further subdivided into microporous, mesoporous and macroporous membranes as discussed earlier. Of these, only microporous and dense membranes can be regarded as being permselective (selective permeability), although macroporous membranes are often used as supports for microporous and dense membranes to accommodate high pressure gradients.^[11]^

For porous membranes, three types of transport mechanisms are possible: bulk flow through membrane pores, diffusion through pores, and restricted diffusion through pores (also called *sieving*). Figure 4 illustrates these different types of transport in porous membranes.

Bulk flow through membrane pores occurs when there is an applied TMP and the membrane pore diameter is large in comparison to the particle size of the solute(s)‐this scenario allows for no selective permeability in transport as there is no restriction on the solute(s) being transferred and is undesirable. When gradients besides pressure, such as activity, fugacity, concentration, etc., are applied across the membrane barrier, selective permeation can be achieved, which leads to diffusion through the membrane pores. Restricted diffusion occurs when the membrane pore sizes are on the same order of molecular size for the solutes of interest, resulting in separation that is improved over pore diffusion alone. This transport phenomenon, also referred to as sieving, is highly desired in filtration applications where size‐based selective permeability is important.^[11]^

### 4.3 Membranes in Bioprocessing

Membrane‐based separations were known for over a century, yet, their industrial‐ scale adoption has only occurred over the last 70 years or so, beginning with the use of porous fluorocarbon membranes in separating ^235^UF_6_ from ^238^UF_6_.[^11^, ^87^] The first successful desalination of seawater to produce potable water utilized RO membranes cast from cellulose acetate.[^11^, ^88^] From these early ventures, MS have come to inhabit a wide variety of chemical processes and continue to be adopted across industries.

Some of the earliest membrane separation applications in biorefining were tested as early as the 1960s in pulp mills, with an RO system installed in a semi‐chemical pulp mill in Green Bay, WI, to concentrate spent pulping liquors and generate white liquor.[^89^, ^90^] The following sections show case some of the literature that has focused on the use of UF membranes in recovery and purification of the biopolymers, lignin and hemicelluloses, and applications in fermentation processes.

#### 4.3.1 Process Lignin Separation

Attention over the last twenty years has shifted to fractionating cooking liquors from kraft and sulfite mills to purify the lignin and recover solubilized carbohydrates. The Borregaard pulp mill in Norway commercialized the adoption of MS to purify lignosulfonates prior to its conversion into vanillin.^[89]^ Researchers have experimented with polymeric, as well as ceramic membranes for application such as lignin recovery from black liquors. He, et al. provide a good review of these applications; owing to its dominant stature in pulping globally, the majority of works cited have investigated the UF of kraft black liquors.^[74]^ Arkell et al.^[91]^ report on the ceramic membrane UF of softwood kraft black liquor and its impact on improving subsequent NF. Toledano et al.^[92]^ report on the UF of alkaline lignin from Chinese silver grass (*Miscanthus sinensis*) using tubular ceramic membranes (made of TiO_2_) over a range of MWCOs (5–15 kDa). Keyoumu et al.^[93]^ report on the fractionation of hardwood and softwood kraft black liquors using ZrO_2_‐coated ceramic membranes with MWCOs of 1, 5, and 15 kDa.

#### 4.3.2 Hemicelluloses Separation

Hemicelluloses recovery from pulping process waters using UF has also been studied along the same lines of maximizing materials recovery from pulping effluents.^[74]^ UF has previously been considered as a way to concentrate biopolymers such as polysaccharides over other techniques such as centrifugation and precipitation, primarily because the former can be carried out at room temperature and has greater relative scalability. From the perspective of generating concentrated extracts of hemicelluloses, MS affords a convenient way to separate hemicellulosic oligomers based on molecular weight while preventing their degradation or chemical modification as the unit operation can be undertaken at ambient conditions. The latter aspect also makes the technique attractive for implementation in larger‐scale settings as process conditions are not likely to be limiting.

Jorda et al.^[94]^ studied the effect of filtering arabinoxylan and rhamnogalacturonan solutions and found that a hollow fiber membrane with an MWCO of 6 kDa provided the best results for arabinoxylan purification. Schlesinger, et al.^[95]^ utilized UF and NF to separate and retain near‐quantitative hemicellulose oligomers at sizes over 1,000 g/mol. When it comes to preserving and retaining hemicelluloses, research efforts have shown progress using MS techniques to separate galactoglucomannan oligomers from pulping liquors.^[96]^ Wang, et al.^[97]^ performed the retention of hemicelluloses in a different fashion by agglomerating the various interfering lignin macromolecular entities and then destabilizing them to separate these from hemicellulose‐derived oligosaccharides. Similarly, Krawczyk and Jonsson^[98]^ reported on the MF of thermomechanical pulp (TMP) liquor to yield ~50 % galactoglucomannan using various pore size microfilters. Wallmo, et al.^[99]^ studied the role that kraft black liquor hemicelluloses play in the separation of lignin using a combination of precipitation and membrane filtration. Li, et al.^[100]^ explored extraction of hemicelluloses from a kraft dissolving pulp preparation of three different hardwood chips in an acidic pre‐hydrolysis stage prior to pulping. Recently, the technoeconomics of the concentration of biomass sugars using MS was modeled and compared to falling‐film evaporation.^[101]^ A two‐stage approach combining preconcentration (using MS), followed by evaporation was found to be the least energy‐intensive strategy for concentrating carbohydrate streams containing glucose and xylose at 35 g/L to 600 g/L. The authors suggest that this does result in greater capital expenses for the MS system.

#### 4.3.3 Membranes in Fermentation

Research using membranes in bioprocessing has also explored the removal of fermentation inhibitors such as weak acids, furans, and phenolic compounds from fermentation broths in cellulosic ethanol production processes. Many of these inhibitors, such as acetic acid,[^102^, ^103^, ^104^] furfural,^[104]^ vanillin,^[74]^ etc., are also valuable chemicals that are attractive for upgrading. In a similar vein, studies have explored the use of membrane processes in the recovery of enzymes (cellulases), which are estimated to account for 20 % of the total cost of ethanol production.^[105]^ Estimated cost savings due to cellulase and cellobiohydrolase recovery and recycling in an ammonia fiber expansion (AFEX) process ranged between 15 and 50 % in comparison to processes in which recovery was absent.^[106]^ When it comes to the finished product, cellulosic (or non‐cellulosic) ethanol, membranes may have a role to play in accomplishing the water reduction at the final stage. Traditional distillation is unable to accomplish the dehydration of ethanol >99 % (by volume) and requires techniques such as azeotropic distillation or molecular sieve adsorption to break the water‐ethanol azeotrope. However, these techniques are energy‐intensive and add to process costs, which is where membrane pervaporation is utilized as an energy‐efficient unit operation. Ethanol dehydration costs using membrane pervaporation processes were calculated to be less than half of those incurred using other techniques, in particular distillation.[^107^, ^108^] Membranes have found their way into anaerobic digestion processes, as well, making use of wastewaters from bioprocessing operations, kraft evaporator condensate, manure, municipal wastewaters, etc., where they were shown to be effective in reducing the chemical oxygen demand (COD), and improved biogas production due to prevention of biomass loss from the bioreactor.[^74^, ^109^]

#### 4.3.4 Biopharmaceuticals

Nearly all monoclonal antibodies production is considered to employ MS during processing, including initial clarification of the cell culture fluid, and UF for concentration and buffer exchange, among others.^[110]^ Typical biotechnological processes can be considered to consist of anywhere between 10 to 20 stages of MS.^[111]^ More recently, increased interest in continuous bioprocessing (over batch mode) in the biotechnological industry to reduce costs and improve productivity and product quality has led to developments in MS technology.^[110]^

#### 4.3.5 Gas Separations

Industrial separations of gases are currently dominated by technologies such as cryogenic distillation, pressure and temperature swing adsorption (PSA, TSA), and chemical absorption, depending on the required purity and recovery, flow rate, and capital costs.^[112]^ Membranes already see widespread application in the natural gas industry for separations, primarily for CO_2_ removal. Since raw natural gas contains a host of undesirable constituents (such as CO_2_, N_2_, H_2_S, He, and other hydrocarbons), it is purified using ‘gas sweetening’ to meet pipeline transmission quality standards. This purification also has the effect of improving the calorific value, decrease gas transportation volume, reduce pollution, and prevent the corrosion of pipelines.^[113]^ While techniques such as amine absorption is one of the most developed, alternatives such as MS are gaining ground in the industry, with cited advantages such as: greater separation efficiency, faster separation and operational simplicity, and space economy.^[113]^ Advancements in MS from this industry can be borrowed to biomass processing when dealing with gas separations, particularly in the context of energy transition and decarbonization—this includes biomethane and hydrogen production and carbon dioxide removal (CDR).

In biogas upgrading, methane must be separated from the other constituents in biogas (namely, CO_2_, H_2_S, etc.) along with trace contaminants. Separation of these constituents from the biogas mixture enables the resulting biomethane to have greater calorific value and the reduction of emissions, similar to that of natural gas sweetening.^[114]^ While water scrubbing was historically the preferred method for biomethane plants due to its relative simplicity, advancements in technology have made other separation methods such as chemical scrubbing, temperature and pressure swing adsorption, and membrane separations more cost‐effective—leading to increased adoption.^[114]^ Basu, et. al.^[115]^ reviewed the application of commercially‐available polymeric membranes for biogas separations, with particular emphasis on the selectivity of CH_4_ and CO_2_ separations. They discuss chemical modifications (side chain and backbone modifications) of commercial membranes by introducing bulky functional groups or replacing flexible bonds (e. g., ‐SiO‐ linkages) with tougher bonds (‐SiCH_2_‐ linkages) to increase CH_4_‐CO_2_ selectivity, glass‐rubber transition temperature, and chain packing density. Table 2 documents recent literature pertaining to applications in bioprocessing.

**Table 2:** Review of recent literature on MS in biorefining.

Product/Compounds/ Applications	MS Type(s)	References
Hemicelluloses/ Pentoses	ED, UF	Purification of pentoses from hemicellulose hydrolysates and recovery of sulfuric acid following UF^[116]^
	NF	Removal of phenolics from wheat straw hydrolysates for detoxification^[117]^
Lignin	UF	Sequential UF of kraft lignin from black liquor dissolved in acetic acid to produce fractions with lower polydispersity^[118]^
	UF	Sequential UF of E. globulus derived kraft lignin showed reduced contamination using lower MWCO membrane^[119]^
	NF	Carbon nanotube (CNT)/Psf NF mixed matrix membranes for rejection of kraft lignins^[120]^
		Fractionation of low‐molecular weight (<1 kDa) lignin moieties from alkaline pretreated corn stover liquor^[121]^
Polyphenols	UF, NF	Separation of polyphenols and anthocyanins form pomegranate juice using flat‐sheet membranes^[122]^
	MF, UF, NF, RO	NF membranes were found to be selective for separation of polypheonls from winery and olive mill wastes^[123]^
Proteins		Use of PAM membrane to separate lactoferrin (with lactic acid and probiotics) from whey to propose a dairy biorefinery^[124]^
	NF	Simulation of ethanol and ‘green protein’ production using NF^[125]^
	MF	MF of lipids and proteins as model mixtures of microalgal lysates^[126]^
	UF	UF of microalgal water soluble proteins from chlorophyll^[127]^
Xylitol		Purification of xylitol obtained from fermentation of sugarcane bagasse hemicellulose hydrolysate^[128]^
	NF	Separation of xylitol from arabinose, xylose solutions using PES membrane,^[129]^ and using NF membrane blended with Pluronic f127 hydrogel^[130]^
Acetic Acid	NF, RO	Comparison of NF, RO and ion‐exchange resins for acetic acid separations from aqueous solutions^[131]^
		Polymer‐ceramic hybrid membranes for separation of acetic acid from water^[132]^
Furans	NF	NF following hydrothermal pretreatment of transgenic sugarcane bagasse for recovery of hydroxymethylfurfural (HMF) and furfural^[133]^
	NF, RO	Removal of furfural^[134]^ and HMF^[135]^ from synthetic solutions—comparisons between using NF and RO
SAF		Volatile fatty acid (VFA) production via membrane‐assisted arrested methanogenesis of wastewater^[136]^
		Reduction of microbial toxicity by removal of biofuel from reactor using membranes^[137]^
ILs and/or DES	MF	Solvent recycling of ILs and DES using MF from pretreatment of a variety of biomass^[138]^
	ED	DES solvent recovery using bipolar membrane electrodialysis^[139]^
	NF	IL solvent regeneration by separating saccharides using NF membranes^[140]^
Biogas	RO	Removal of H_2_S and CO_2_ using water‐swollen composite RO membranes[^141^, ^142^]
		Industrial scale case study on production of food‐grade CO_2_ from biogas using polyimide commercial SEPURAN® membrane modules^[143]^

From the above examples, membrane‐based separation processes afford many advantages, chief among which are lower operating cost and energy requirements, and flexibility with regards to operating conditions and feedstock. This would make membranes an ideal separation strategy for biorefining applications where dilute streams are the norm, and solutes are often tough to separate from one another due to similar physicochemical properties. Depending on the processing conditions employed, large macromolecules are frequently encountered and need to be concentrated. Using the classification available by pore size, the unit operation of UF would be the most applicable for separations involving macromolecules, which underutilized biomass streams of interest, lignin and hemicellulose, comprise. MS also come with a host of challenges that need to be accounted for when designing separation strategies. Some of the drawbacks associated with using MS are discussed below.

### 4.4 Challenges in MS

#### 4.4.1 Membrane Fouling

An important operational consideration associated with MS is membrane fouling, a concern that is pervasively associated with this technology. Fouling of membranes is typically thought to be associated with adsorptive interactions between the solutes and the membrane surface‐in the case of NF of water, and adsorptive interactions which result in the formation of a hydrophobic barrier on the membrane surface, leading to a reduction in filtrate flux. In general, the adoption of inorganic membranes is thought to reduce the potential for fouling as compared to organic polymeric membranes.^[77]^ Fouling can occur due to colloids, inorganic ions, biologically active organisms, and biofilms, or other factors, depending on the processing conditions and feedstock. Addressing fouling issues requires additional resources and the need for pretreatment where applicable, results in operational downtime, and adds to processing costs due to cleaning needed, and reduces the lifetimes of the membranes.^[144]^

While membrane fouling is typically identified by monitoring the flux (decrease) or the pressure (increase) over time, this information provides limited insight into the nature, amount, and location of fouling. Recent research on more strategic treatment of membrane fouling corresponds to the implementation of real‐time, in situ monitoring tools such as spectroscopic, acoustic, controlled current techniques, etc.^[145]^ While these have not been widely implemented in commercial settings, advancements in this field can allow for more efficient management of membrane fouling challenges.

#### 4.4.2 Process Development, Materials, and Sustainability

The manufacture of membranes (polymeric/inorganic) requires the use of solvents such as n‐Methylpyrrolidone (NMP), dimethylacetamide (DMAc), dimethylformamide (DMF), dimethylacetamide (DMA), tetrahydrofuran (THF), hexane, chloroform, etc.^[146]^ Polymeric membranes utilize fossil‐derived precursors such as poly(vinylidene fluoride) (PVDF), polyethersulfone (PES), polysulfone (PSf), polyacrylonitrile (PAN), polyether ether ketone (PEEK), etc.^[146]^ Long‐term reliance on such toxic and non‐renewable materials and solvents can be antithetical for the future of green biorefineries which are predicated on increased sustainability and fewer environmental burdens and harms. The commercial manufacture of membranes is undergoing a transformation towards increasing process sustainability and overall life cycle.^[147]^ Challenges associated with the production of membranes (e. g., energy consumption, waste generation, etc.) are being scrutinized with a green chemistry and sustainability approach.[^146^, ^148^]

The choice of solvents currently used in membrane manufacture include dimethylformamide (DMF), N‐methylpyrrolidone, and N,N‐dimethylacetamide, dimethylacetamide (DMAc), dimethylacetamide (DMA), tetrahydrofuran (THF), hexane, chloroform, etc., which are associated with concerns of toxicity,[^146^, ^147^, ^149^] and correspondingly, environmental sustainability.^[150]^ This also ties in with the need to move away from fossil‐based sources of membrane manufacturing solvents to renewable sources. The latter includes solvents such as dimethylsulfoxide (DMSO), supercritical CO_2_, ionic liquids, etc.^[147]^ While much of this development is occurring in the realm of water applications (e. g., wastewater treatment, desalination, etc.), it can be reasonably anticipated that these will eventually filter through into biorefining applications as the field matures and is better able to integrate MS.

#### 4.4.3 Concentration

In specific applications, implementing membrane separation technology can give rise to additional concerns. For example, using NF for wastewater treatment results in a concentrated contaminant stream that requires disposal. Similarly, using RO for drinking water purification also delivers a concentrated waste stream that requires handling while also rejecting large volumes of water compared to the amount treated. This result could be of significant concern regarding low yield in situations where drinking water resources are dwindling, as in the case of groundwater.^[144]^ In chemical refining, challenges arising out of solvent behavior are experienced even with aqueous solutions where extensive swelling can give rise to a loss of selectivity. Addressing some of these challenges requires a deeper understanding of membrane‐solvent interactions and modeling approaches for predicting membrane behavior.[^144^, ^151^]

#### 4.4.4 Narrow Range of Operability

As discussed previously, the application of polymeric membranes is limited to processes that usually operate at mild temperatures and chemically inert mixtures. That said, it has been suggested that the operable range of polymeric membranes may be extended using high‐performance polymers such as polyimides, polyether ether ketone (PEEK), etc.^[147]^ The option of utilizing inorganic membranes for conditions outside of this offers greater flexibility with processing conditions. Precursors for the manufacture of ceramic membranes such as α‐alumina, γ‐alumina, zirconia, etc., are much more expensive, and this translates into greater installation and operational costs as a result;[^152^, ^153^] although inexpensive precursors (e. g., kaolin, quartz, feldspar, etc.) for the manufacture of ceramic membranes are being explored, the research is still in infancy.

## 5 Current Status and Future Outlook

The global market size for MS technologies in 2021 was estimated at $22.1 billion, with future growth through 2030 expected at an annual rate of 12 %. This growth was noted to be primarily driven by increasing governmental regulations around food safety and pollution prevention, increased adoption of MS by the food and beverage industries, and new opportunities for the separation of chemical compounds by MS, among others.^[154]^ Some of the challenges and prospects in the future development of MS were identified^[155]^ to lie in the areas of membrane construction materials (polymeric and inorganic membranes), membrane module production technologies (including the use of green solvents such as water and supercritical CO_2_, application of 3D printing technologies, etc.), and new industrial applications (biorefineries).

As with other fields that are impacted by the advent of artificial intelligence (AI) and machine learning (ML), the future of MS is similarly thought to be influenced by ML technologies, particularly in regard to membrane material design and development.^[156]^ Recent research[^157^, ^158^] into tuning the pore structure of membranes based on ambient conditions such as light, pH, temperature, ionic strength, electric and magnetic fields, etc., has led to the development of smart membranes with stimuli‐responsive capabilities. These advancements may eventually find their way into biorefining where the product streams and separation requirements can change in real‐time. This ties into the observation that MS applications (both commercial and R&D) appear to lag behind those in water and wastewater treatment. This can delay their implementation in biorefining as technological improvements from the water purification sphere first need to be adapted to biomass and their product streams. This highlights the need for pioneering research on MS applications in biorefining rather than as an afterthought borrowed from other areas of applications.

To overcome some of the inherent challenges in MS, one of the proposed approaches is the PI framework which seeks to improve energy efficiency by the replacement of energy‐intensive separation strategies.[^159^, ^160^] As introduced earlier under reactive separations, PI can also reduce the generation of wastes and improve scalability by incorporating concepts such as miniaturization, integrated optimization and control, shorter diffusion pathways, and reduction in the number of devices by singularization of the apparatus, which is critical for biorefineries.[^50^, ^161^] While focusing on PI seems to be a promising approach, R&D efforts are still needed which can reduce costs and the large footprint associated with membranes, particularly inorganic membrane applications.^[162]^

Finally, as there is increased momentum in the transition from conventional petrochemical refineries to biorefineries (and e‐refineries) the focus is on advanced separation techniques, such as MS, to enable energy‐efficient capacity building. That said, it is imperative to ‘hybridize’ existing separation systems (such as distillation) with advanced separations due to inherent advantages of capital costs and product purity and recovery in the former.[^73^, ^163^] This ‘hybridization’ is likely to resemble a gradual transition away from traditional thermal separation processes to advanced separations such as MS, as incremental capacity is added to facilitate the biorefineries of the future.^[73]^

## 6 Conclusions

To conclude, MS have been adopted in various applications and have been embraced by diverse industries ranging from chemical processing and refining to water treatment. Despite the challenges posed by membranes, their flexibility and lower‐cost operation are attractive for emerging industries such as biorefineries. Their advantages make them an ideal candidate for pursuing separations of biomass processing streams. Research on the development of integrated biorefineries that process lignocellulosic feedstock needs to refocus on the challenges encountered in separating bioprocessing streams, and filtration membranes offer advantages that make them appealing. As the review shows, MS have come a long way from niche applications in specific industries to being the focus of federal research initiatives for future biorefineries, and have an outsized role to play in addressing the climate crisis.

## Conflict of Interests

The authors declare no conflict of interest.

7

## Biographical Information

*Dr. Anurag Mandalika is an assistant research professor at the Center for Energy Studies at Louisiana State University. He earned his PhD and MS in Biological Systems Engineering from the University of Wisconsin‐Madison, and his BS in Biosystems Engineering from Clemson University. His current research is focused on understanding the potential for biohydrogen and sustainable biofuels by integrating biomass wastes and residues based on concepts of green chemistry and the circular bioeconomy targeting industrial decarbonization and energy resiliency*.

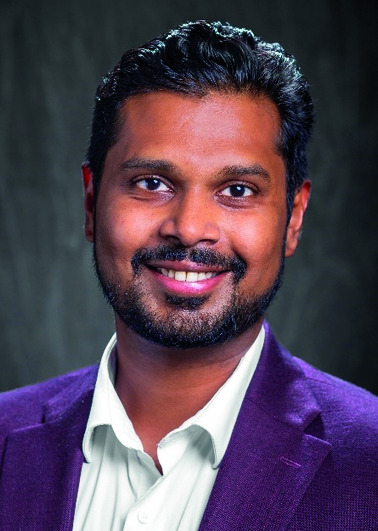

## Biographical Information

*Dr. Troy Runge is a professor of Biological Systems Engineering and the College of Agricultural and Life Sciences Associate Dean for Research at the University of Wisconsin Madison. He has over 30 years of experience in biomass processing and separation technologies. He obtained his PhD from the Institute of Paper Science and Technology at the Georgia Institute of Technology. His current research focuses on biomass deconstruction, separation and system analysis*.

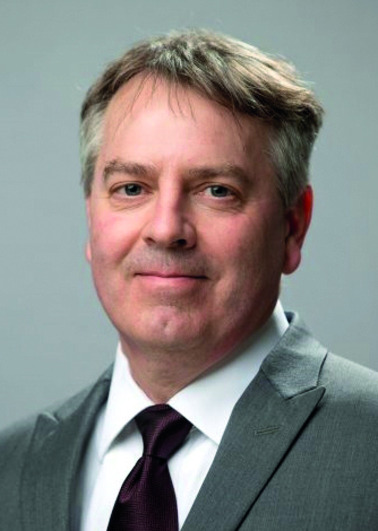

## Biographical Information

*Dr. Arthur Ragauskas held the first Fulbright Chair in Alternative Energy and is a Fellow of the American Association for the Advancement of Science, the International Academy of Wood Science, and TAPPI. In 2014, he assumed a Governor's Chair for Biorefining based in the University of Tennessee's Department of Chemical and Biomolecular Engineering, with a complementary appointment in the UT Institute of Agriculture's Department of Forestry, Wildlife, and Fisheries and serves in the Energy and Environmental Sciences Directorate, Biosciences Division, at ORNL. His research program is directed at understanding and exploiting innovative sustainable bioresources for the circular economy. Dr. Ragauskas was the major advisor for Dr. Runge, who was, in turn, the major advisor for Dr. Mandalika. This work represents a collaboration spanning three generations of research into biorefining*.

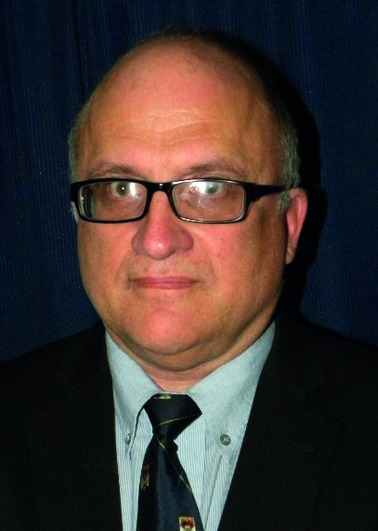

## Data Availability

Data sharing is not applicable to this article as no new data were created or analyzed in this study.

## References

[1] B. Kamm , M. Kamm , Appl. Microbiol. Biotechnol. 2004, 64(2), 137–145. DOI: 10.1007/s00253-003-1537-7. 14749903

[2] B. Kamm , P. R. Gruber , M. Kamm , Biorefineries-Industrial Processes and Products, Wiley-VCH, Weinheim 2006.

[3] S. K. O. Maity , Renew. Sustain. Energy Rev. 2015, 43, 1427–1445. 10.1016/j.rser.2014.11.092.

[4] A. A. Kiss , J.-P. Lange , B. Schuur , D. W. F. Brilman , A. G. J. van der Ham , S. R. A. Kersten , Biomass Bioenergy 2016, 95, 296–309. 10.1016/j.biombioe.2016.05.021.

[5] J. H. Clark , J. Chem. Technol. Biotechnol. 2007, 82(7), 603–609. DOI: 10.1002/jctb.1710 (acccessed 2020/05/25).

[6] G. W. Huber , S. Iborra , A. Corma , ChemInform 2006, 37(52), 4044–4098. DOI: 10.1002/chin.200652240. 16967928

[7] M. Fatih Demirbas , Appl. Energy 2009, 86, S151–S161. 10.1016/j.apenergy.2009.04.043.

[8] H.-J. Huang , S. Ramaswamy , U. W. Tschirner , B. V. Ramarao , Sep. Purif. Technol. 2008, 62(1), 1–21. 10.1016/j.seppur.2007.12.011.

[9] M. Sagehashi , T. Nomura , H. Shishido , A. Sakoda , Bioresour. Technol. 2007, 98(10), 2018–2026. 10.1016/j.biortech.2006.08.022. 17070042

[10] A. J. Ragauskas , C. K. Williams , B. H. Davison , G. Britovsek , J. Cairney , C. A. Eckert , W. J. Frederick , J. P. Hallett , D. J. Leak , C. L. Liotta , et al., Science 2006, 311(5760), 484. DOI: 10.1126/science.1114736. 16439654

[11] J. D. Seader , E. J. Henley , D. K. Roper , Separation Process Principles, Wiley, New York 1998.

[12] K. Srirangan , L. Akawi , M. Moo-Young , C. P. Chou , Appl. Energy 2012, 100, 172–186. 10.1016/j.apenergy.2012.05.012.

[13] U.S. Department of EnergyM. H. Langholtz (Lead), *2023 Billion-Ton Report: An Assessment of U.S. Renewable Carbon Resources*, Oak Ridge National Laboratory. ORNL/SPR-2024/3103. **2024**. DOI: 10.23720/BT2023/2316165.

[14] J. A. Okolie , S. Nanda , A. K. Dalai , J. A. Kozinski , Waste Biomass Valorization 2021, 12(5), 2145–2169. DOI: 10.1007/s12649-020-01123-0.

[15] A. Thygesen , J. Oddershede , H. Lilholt , A. B. Thomsen , K. Ståhl , Cellulose 2005, 12(6), 563–576. DOI: 10.1007/s10570-005-9001-8.

[16] T. E. Timell , Wood Hemicelluloses: Part II. In Advances in Carbohydrate Chemistry, Volume 20 (Ed: L. W. Melville ), Academic Press, 1965, pp. 409–483.

[17] S. Suzuki , Y. Suzuki , N. Yamamoto , T. Hattori , M. Sakamoto , T. Umezawa , Plant Biotechnol. (Tokyo) 2009, 26(3), 337–340.

[18] A. V. Tran , R. P. Chambers , Enzyme Microb. Technol. 1986, 8(7), 439–444. DOI: 10.1016/0141-0229(86)90154-7.

[19] M. S. Tunc , A. R. P. van Heiningen , Ind. Eng. Chem. Res. 2008, 47(18), 7031–7037. DOI: 10.1021/ie8007105 (accessed 2012/07/03).

[20] L. Turner, International Affairs (Royal Institute of International Affairs 1944–) **1974**, *50*(*3*), 404–415.

[21] J. van Dyk, M. Keyser, M. Coertzen, *Sasol's unique position in syngas production from South African coal sources using Sasol-Lurgi fixed bed dry bottom gasifiers*. In World of Coal Ash Conference, Lexington, Kentucky **2005**.

[22] S. Besseau , L. Hoffmann , P. Geoffroy , C. Lapierre , B. Pollet , M. Legrand , Plant Cell 2007, 19(1), 148. DOI: 10.1105/tpc.106.044495. 17237352 PMC1820963

[23] A. Scalbert , Phytochemistry 1991, 30(12), 3875–3883. 10.1016/0031-9422(91)83426-L.

[24] A. Belščak-Cvitanović , K. Durgo , A. Huđek , V. Bačun-Družina , D. Komes , Polyphenols: Properties, Recovery, and Applications, (Ed: C. M. Galanakis ), W. Publishing, 2018, 3–44.

[25] S. Quideau , D. Deffieux , C. Douat-Casassus , L. Pouységu , Angew. Chem. Int. Ed. 2011, 50(3), 586–621. DOI: 10.1002/anie.201000044 (acccessed 2018/08/02). 21226137

[26] S. V. Vassilev , D. Baxter , L. K. Andersen , C. G. Vassileva , Fuel 2013, 105, 40–76. 10.1016/j.fuel.2012.09.041.

[27] A. Mandalika , T. Runge , Green Chem. 2012, 14(11), 3175–3184.

[28] A. Mandalika , L. Qin , T. K. Sato , T. Runge , Green Chem. 2014, 16(5), 2480–2489.

[29] M. Fache , B. Boutevin , S. Caillol , ACS Sustain. Chem. Eng. 2016, 4(1), 35–46. DOI: 10.1021/acssuschemeng.5b01344.

[30] N. Singh , R. R. Singhania , P. S. Nigam , C.-D. Dong , A. K. Patel , M. Puri , Bioresour. Technol. 2022, 344, 126415. 10.1016/j.biortech.2021.126415. 34838977

[31] EERE. Integrated Biorefineries. US Department of Energy, **2024**. https://www.energy.gov/eere/bioenergy/integrated-biorefineries(accessed).

[32] B. Burmahl , Separations Technology Critical To Converting Biomass To Low-Carbon Fuel, Argonne National Laboratory, Lemont, IL 2023.

[33] M. F. S. Dubreuil , K. Servaes , D. Ormerod , D. Van Houtven , W. Porto-Carrero , P. Vandezande , G. Vanermen , A. Buekenhoudt , Sep. Purif. Technol. 2017, 178, 56–65. 10.1016/j.seppur.2016.12.033.

[34] J. Bausa , G. Tsatsaronis , Comput. Chem. Eng. 2001, 25(2), 359–370. 10.1016/S0098-1354(00)00664-5.

[35] M. Jobson , Distillation, (Eds: A. Górak , E. Sorensen ), Academic Press, 2014, pp. 225–270.

[36] Y. H. Kim , Korean J. Chem. Eng. 2016, 33(9), 2513–2521. DOI: 10.1007/s11814-016-0124-4.

[37] F. M. Antony , D. Pal , K. Wasewar , Phys. Sci. Rev. 2021, 6(4), 20180065.

[38] H. Ullah , C. D. Wilfred , M. S. Shaharun , Sep. Sci. Technol. 2019, 54(4), 559–579. DOI: 10.1080/01496395.2018.1505913.

[39] A. M. da Costa Lopes , R. M. Łukasik , ChemSusChem 2018, 11(6), 1099–1107. DOI: 10.1002/cssc.201702231(acccessed 2024/07/31). 29400913

[40] Y. Wang , K. H. Kim , K. Jeong , N.-K. Kim , C. G. Yoo , Curr. Opin. Green Sustain. Chem. 2021, 27, 100396. 10.1016/j.cogsc.2020.100396.

[41] A. Bjelić , B. Hočevar , M. Grilc , U. Novak , B. Likozar , Rev. Chem. Eng. 2022, 38(3), 243–272.

[42] V. Sharma , M.-L. Tsai , C.-W. Chen , P.-P. Sun , A. K. Patel , R. R. Singhania , P. Nargotra , C.-D. Dong , Bioresour. Technol. 2022, 360, 127631. 10.1016/j.biortech.2022.127631. 35850394

[43] A. Isci , M. Kaltschmitt , Biomass Convers. Biorefin. 2022, 12(1), 197–226. DOI: 10.1007/s13399-021-01860-9.

[44] K. Schute , C. Detoni , A. Kann , O. Jung , R. Palkovits , M. Rose , ACS Sustain. Chem. Eng. 2016, 4(11), 5921–5928. DOI: 10.1021/acssuschemeng.6b00096.

[45] B. M. Travália , M. B. Soares Forte , J. Chem. Eng. Data 2020, 65(9), 4503–4511. DOI: 10.1021/acs.jced.0c00340.

[46] N. A. S. Din , S. J. Lim , M. Y. Maskat , S. A. Mutalib , N. A. M. Zaini , Bioresour. Bioprocess. 2021, 8(1), 31. DOI: 10.1186/s40643-021-00384-4. 38650212 PMC10991309

[47] J. Ran , L. Wu , Y. He , Z. Yang , Y. Wang , C. Jiang , L. Ge , E. Bakangura , T. Xu , J. Membr. Sci. 2017, 522, 267–291. 10.1016/j.memsci.2016.09.033.

[48] R. Krishna , Chem. Eng. Sci. 2002, 57(9), 1491–1504. 10.1016/S0009-2509(02)00020-9.

[49] W. A. Leet , S. Kulprathipanja , Reactive Separation Processes, CRC Press, 2019, pp. 1–17.

[50] A. Stankiewicz , Chem. Eng. Process. Process Intensif. 2003, 42(3), 137–144. 10.1016/S0255-2701(02)00084-3.

[51] N. Boon-anuwat , W. Kiatkittipong , F. Aiouache , S. Assabumrungrat , Chem. Eng. Process. Process Intensif. 2015, 92, 33–44. 10.1016/j.cep.2015.03.025.

[52] T. Poddar , A. Jagannath , A. Almansoori , Appl. Energy 2017, 185, 985–997. 10.1016/j.apenergy.2015.12.054.

[53] M. McVey , Y. Elkasabi , D. Ciolkosz , Biomass Convers. Biorefin. 2020, 10(1), 15–23. DOI: 10.1007/s13399-019-00409-1.

[54] M. A. M. Hussain , P. H. Pfromm , Sep. Sci. Technol. 2013, 48(10), 1518–1528. DOI: 10.1080/01496395.2013.766211.

[55] L. C. Nhien , N. V. D. Long , M. Lee , Energy Convers. Manage. 2017, 141, 367–377. 10.1016/j.enconman.2016.09.077.

[56] J. Zhang , C. Lei , G. Liu , Y. Bao , V. Balan , J. Bao , ACS Sustain. Chem. Eng. 2017, 5(12), 11676–11685. DOI: 10.1021/acssuschemeng.7b03084.

[57] M. Gryta , Sep. Purif. Technol. 2001, 24(1), 283–296. 10.1016/S1383-5866(01)00132-0.

[58] G. Lewandowicz , W. Białas , B. Marczewski , D. Szymanowska , J. Membr. Sci. 2011, 375(1), 212–219. 10.1016/j.memsci.2011.03.045.

[59] A. Singh , G. P. Rangaiah , J. Chem. Technol. Biotechnol. 2019, 94(4), 1041–1056. DOI: 10.1002/jctb.5851 (acccessed 2024/08/19).

[60] R. Palacios-Bereche , A. V. Ensinas , M. Modesto , S. A. Nebra , Energy 2015, 82, 512–523. 10.1016/j.energy.2015.01.062.

[61] L. C. B. A. Bessa , M. C. Ferreira , E. A. C. Batista , A. J. A. Meirelles , Energy 2013, 63, 1–9. 10.1016/j.energy.2013.10.006.

[62] J. Li , C. Wang , Z. Yang , J. Anal. Appl. Pyrolysis 2010, 89(2), 218–224. 10.1016/j.jaap.2010.08.004.

[63] Y. Hou , Z. Feng , J. R. Sossa Cuellar , W. Wu , Pure Appl. Chem. 2020, 92(10), 1717–1731.

[64] J. Tan , Z. Sun , H. Huang , G. Zhou , S. Xie , Desalination 2023, 564, 116790. 10.1016/j.desal.2023.116790.

[65] A. Luiz , D. D. McClure , K. Lim , G. Leslie , H. G. L. Coster , G. W. Barton , J. M. Kavanagh , Desalination 2017, 415, 20–28. 10.1016/j.desal.2017.02.023.

[66] N. A. M. Zaini , A. Chatzifragkou , V. Tverezovskiy , D. Charalampopoulos , Biochem. Eng. J. 2019, 150, 107265. 10.1016/j.bej.2019.107265.

[67] C. H. Bühlmann , B. S. Mickan , S. Tait , P. A. Bahri , Chem. Eng. J. 2022, 431, 133243. 10.1016/j.cej.2021.133243.

[68] C. González-Navarrete , E. Sánchez-Ramírez , C. Ramírez-Márquez , S. Hernández , E. Cossío-Vargas , J. G. Segovia-Hernández , Ind. Eng. Chem. Res. 2022, 61(1), 621–637. DOI: 10.1021/acs.iecr.1c04050.

[69] M. Lomelí-Rodríguez , M. Rivera-Toledo , J. A. López-Sánchez , Ind. Eng. Chem. Res. 2017, 56(11), 3017–3032. DOI: 10.1021/acs.iecr.6b04806.

[70] M. Muharja , R. F. Darmayanti , B. A. Fachri , B. Palupi , I. Rahmawati , M. F. Rizkiana , H. W. Amini , D. K. Y. Putri , F. A. Setiawan , M. Asrofi , et al., Bioresour. Technol. Rep. 2023, 21, 101298. 10.1016/j.biteb.2022.101298.

[71] P. K. T. Liu , M. Sahimi , T. T. Tsotsis , Curr. Opin. Chem. Eng. 2012, 1(3), 342–351. 10.1016/j.coche.2012.06.001.

[72] G. S. Murthy , S. Sridhar , M. S. Sunder , B. Shankaraiah , M. Ramakrishna , Sep. Purif. Technol. 2005, 44(3), 205–211. 10.1016/j.seppur.2005.01.007.

[73] R. P. Lively , AIChE J. 2021, 67(7), e17286. DOI: 10.1002/aic.17286 (acccessed 2024/08/13).

[74] Y. He , D. M. Bagley , K. T. Leung , S. N. Liss , B.-Q. Liao , Biotechnol. Adv. 2012, 30(4), 817–858. 10.1016/j.biotechadv.2012.01.015. 22306168

[75] P. A. Wright , Microporous framework solids, Royal Society of Chemistry, 2007.

[76] J. S. Beck , J. C. Vartuli , W. J. Roth , M. E. Leonowicz , C. T. Kresge , K. D. Schmitt , C. T. W. Chu , D. H. Olson , E. W. Sheppard , S. B. McCullen , et al., J. Am. Chem. Soc. 1992, 114(27), 10834–10843. DOI: 10.1021/ja00053a020.

[77] A. Lee , J. W. Elam , S. B. Darling , Environ. Sci. Water Res. Technol. 2016, 2(1), 17–42.

[78] A. Kayvani Fard , G. McKay , A. Buekenhoudt , H. Al Sulaiti , F. Motmans , M. Khraisheh , M. Atieh , Materials (Basel, Switzerland) 2018, 11(1), 74. DOI: 10.3390/ma11010074 PubMed. 29304024 PMC5793572

[79] E. Fontananova , E. Drioli , Comprehensive Membrane Science And Engineering, Newnes (Vol. 1) (Eds: E Drioli, L. Giorno), Elsevier, Oxford, 2010, pp. 109–133.

[80] E. Drioli , C. A. Quist-Jensen , L. Giorno , Molecular Weight Cutoff. In Encyclopedia of Membranes (Eds: E. Drioli , L. Giorno ), Springer Berlin, Heidelberg, 2016, 1326–1327.

[81] R. Singh , Membrane Technology and Engineering for Water Purification (Second Edition) (Ed: R. Singh), Butterworth-Heinemann, 2015, pp. 1–80.

[82] J. Liderfelt , J. Royce , Biopharmaceutical Processing, (Eds: G. Jagschies , E. Lindskog , K. Łącki , P. Galliher ), Elsevier, 2018, pp. 279–293.

[83] E. Alventosa-deLara , S. Barredo-Damas , M. I. Alcaina-Miranda , M. I. Iborra-Clar , J. Hazard. Mater. 2012, 209–210, 492–500. 10.1016/j.jhazmat.2012.01.065. 22326247

[84] B. D. Mitchell , W. M. Deen , J. Membr. Sci. 1984, 19(1), 75–100. 10.1016/S0376-7388(00)80171-4.

[85] K. N. Bourgeous , J. L. Darby , G. Tchobanoglous , Water Res. 2001, 35(1), 77–90. 10.1016/S0043-1354(00)00225-6. 11257896

[86] K. M. Persson , V. Gekas , G. Trägårdh , J. Membr. Sci. 1995, 100(2), 155–162. 10.1016/0376-7388(94)00263-X.

[87] L. E. Applegate , Chem. Eng. (New York, NY) 1984, 91(12), 64–89.

[88] G. G. Havens , D. B. Guy , Chem. Eng. Prog. Syrup. Ser 1968, 64(90), 299–305.

[89] O. Wallberg , A.-S. Jönsson , R. Wimmerstedt , Desalination 2003, 154(2), 187–199. 10.1016/S0011-9164(03)80019-X.

[90] A. J. Wiley , A. C. F. Ammerlaan , G. Dubey , Tappi 1967, 50, 455–460.

[91] A. Arkell , J. Olsson , O. Wallberg , Chem. Eng. Res. Des. 2014, 92(9), 1792–1800. 10.1016/j.cherd.2013.12.018.

[92] A. Toledano , A. García , I. Mondragon , J. Labidi , Sep. Purif. Technol. 2010, 71(1), 38–43. 10.1016/j.seppur.2009.10.024.

[93] A. Keyoumu , R. Sjödahl , G. Henriksson , M. Ek , G. Gellerstedt , M. E. Lindström , Ind. Crops Prod. 2004, 20(2), 143–150.

[94] J. Jorda , P. Marechal , L. Rigal , P. Y. Pontalier , Desalination 2002, 148(1–3), 187–191. 10.1016/S0011-9164(02)00696-3.

[95] R. Schlesinger , G. Götzinger , H. Sixta , A. Friedl , M. Harasek , Desalination 2006, 192(1), 303–314. 10.1016/j.desal.2005.05.031.

[96] M. Al Manasrah , M. Kallioinen , H. Ilvesniemi , M. Mänttäri , Bioresour. Technol. 2012, 114, 375–381. 10.1016/j.biortech.2012.02.014. 22444636

[97] Z. Wang , X. Wang , Y. Fu , Z. Li , F. Zhang , M. Qin , Sep. Purif. Technol. 2015, 145, 1–7. 10.1016/j.seppur.2015.03.001.

[98] H. Krawczyk , A. S. Jönsson , Sep. Purif. Technol. 2011, 79(1), 43–49. 10.1016/j.seppur.2011.03.009.

[99] H. Wallmo , H. Theliander , A.-S. Jönsson , O. Wallberg , K. Lindgren , Nord. Pulp Pap. Res. J. 2009, 24(2), 165–171.

[100] H. Li , A. Saeed , M. S. Jahan , Y. Ni , A. van Heiningen , J. Wood Chem. Technol. 2010, 30(1), 48–60.

[101] D. A. Sievers , J. J. Stickel , N. J. Grundl , L. Tao , Ind. Eng. Chem. Res. 2017, 56(40), 11584–11592. DOI: 10.1021/acs.iecr.7b02178.

[102] B. Han , W. Carvalho , L. Canilha , S. S. da Silva , J. B. Almeida e Silva , J. D. McMillan , S. R. Wickramasinghe , Desalination 2006, 193(1), 361–366. 10.1016/j.desal.2005.07.052.

[103] Y.-H. Weng , H.-J. Wei , T.-Y. Tsai , W.-H. Chen , T.-Y. Wei , W.-S. Hwang , C.-P. Wang , C.-P. Huang , Sep. Purif. Technol. 2009, 67(1), 95–102. 10.1016/j.seppur.2009.03.030.

[104] D. L. Grzenia , D. J. Schell , S. Ranil Wickramsinghe , J. Membr. Sci. 2010, 348(1), 6–12. 10.1016/j.memsci.2009.10.035.

[105] J. S. Knutsen , R. H. Davis , Biotechnology for Fuels and Chemicals: The Twenty–Third Symposium, (Eds: M. Finkelstein , J. D. McMillan , B. H. Davison ), Humana Press, 2002, 1161–1172.

[106] B. Steele , S. Raj , J. Nghiem , M. Stowers , Twenty-Sixth Symposium on Biotechnology for Fuels and Chemicals, (Eds: B. H. Davison , B. R. Evans , M. Finkelstein , J. D. McMillan ), Humana Press, 2005, pp. 901–910.

[107] U. Sander , P. Soukup , J. Membr. Sci. 1988, 36, 463–475. 10.1016/0376-7388(88)80036-X.

[108] W. Kaminski , J. Marszalek , A. Ciolkowska , Chem. Eng. J. 2008, 135(1), 95–102. 10.1016/j.cej.2007.03.017.

[109] E. Jeong, H.-W. Kim, J.-Y. Nam, H.-S. Shin, *Bioresour. Technol*. **2010**, *101*(*1, Supplement*), S7–S12. 10.1016/j.biortech.2009.04.064. 19467588

[110] A. L. Zydney , J. Membr. Sci. 2021, 620, 118804. 10.1016/j.memsci.2020.118804.

[111] A. S. Rathore , A. Shirke , Prepr. Biochem. Biotechnol. 2011, 41(4), 398–421. DOI: 10.1080/10826068.2011.613976. 21967339

[112] Q. Qian , P. A. Asinger , M. J. Lee , G. Han , K. Mizrahi Rodriguez , S. Lin , F. M. Benedetti , A. X. Wu , W. S. Chi , Z. P. Smith , Chem. Rev. 2020, 120(16), 8161–8266. DOI: 10.1021/acs.chemrev.0c00119. 32608973

[113] J. K. Adewole , A. L. Ahmad , S. Ismail , C. P. Leo , Int. J. Greenhouse Gas Control 2013, 17, 46–65. 10.1016/j.ijggc.2013.04.012.

[114] F. M. Baena-Moreno , E. le Saché , L. Pastor-Pérez , T. R. Reina , Environ. Chem. Lett. 2020, 18(5), 1649–1658. DOI: 10.1007/s10311-020-01036-3.

[115] S. Basu , A. L. Khan , A. Cano-Odena , C. Liu , I. F. J. Vankelecom , Chem. Soc. Rev. 2010, 39(2), 750–768. 20111791 10.1039/b817050a

[116] J. Lemaire , C.-L. Blanc , F. Duval , M.-A. Théoleyre , D. Pareau , Sep. Purif. Technol. 2016, 166, 181–186. 10.1016/j.seppur.2016.04.030.

[117] A. Fayet , A. R. S. Teixeira , F. Allais , M. Bouix , M.-L. Lameloise , J. Membr. Sci. 2018, 566, 112–121. 10.1016/j.memsci.2018.08.045.

[118] C. Huang , J. He , R. Narron , Y. Wang , Q. Yong , ACS Sustain. Chem. Eng. 2017, 5(12), 11770–11779. DOI: 10.1021/acssuschemeng.7b03415.

[119] C. A. E. Costa , P. C. R. Pinto , A. E. Rodrigues , Sep. Purif. Technol. 2018, 192, 140–151. 10.1016/j.seppur.2017.09.066.

[120] Manorma , I. Ferreira , P. Alves , M. H. Gil , L. M. Gando-Ferreira , Sep. Purif. Technol. 2021, 260, 118231. 10.1016/j.seppur.2020.118231.

[121] P. O. Saboe , E. G. Tomashek , H. R. Monroe , S. J. Haugen , R. L. Prestangen , N. S. Cleveland , R. M. Happs , J. Miscall , K. J. Ramirez , R. Katahira , et al., Green Chem. 2022, 24(8), 3152–3166. DOI: 10.1039/D2GC00075J.

[122] C. Conidi , A. Cassano , F. Caiazzo , E. Drioli , J. Food Eng. 2017, 195, 1–13. 10.1016/j.jfoodeng.2016.09.017.

[123] P. Tapia-Quirós , M. F. Montenegro-Landívar , M. Reig , X. Vecino , J. Saurina , M. Granados , J. L. Cortina , J. Environ. Manage. 2022, 307, 114555. 10.1016/j.jenvman.2022.114555. 35085965

[124] J. L. Sebastián-Nicolás , L. G. González-Olivares , G. A. Vázquez-Rodríguez , Carlos A. Lucho-Constatino , A. Castañeda-Ovando , A. E. Cruz-Guerrero , Biofuels, Bioprod. Biorefin. 2020, 14 (5), 1010–1027. 10.1002/bbb.2100 (acccessed 2024/08/29).

[125] T. Allan Andrade , C. Ramírez-Márquez , M. Ambye-Jensen , Sep. Purif. Technol. 2023, 327, 124887. 10.1016/j.seppur.2023.124887.

[126] S. Liu , C. Rouquié , M. Frappart , A. Szymczyk , M. Rabiller-Baudry , E. Couallier , Sep. Purif. Technol. 2024, 328, 124985. 10.1016/j.seppur.2023.124985.

[127] C. Safi , G. Olivieri , R. P. Campos , N. Engelen-Smit , W. J. Mulder , L. A. M. van den Broek , L. Sijtsma , Bioresour. Technol. 2017, 225, 151–158. 10.1016/j.biortech.2016.11.068. 27888732

[128] Y. P. Cerceau Alves , F. A. Fernandes Antunes , S. Silverio da Silva , M. B. S. Forte , Food Bioprod. Process. 2021, 125, 79–90. 10.1016/j.fbp.2020.10.005.

[129] K. A. Faneer , R. Rohani , A. W. Mohammad , M. M. Ba-Abbad , Korean J. Chem. Eng. 2017, 34(11), 2944–2957. DOI: 10.1007/s11814-017-0186-y.

[130] K. A. Faneer , R. Rohani , A. W. Mohammad , J. Cleaner Prod. 2018, 171, 995–1005. 10.1016/j.jclepro.2017.10.075.

[131] H. Wu , L. Valentino , S. Riggio , M. Holtzapple , M. Urgun-Demirtas , Sep. Purif. Technol. 2021, 265, 118108. 10.1016/j.seppur.2020.118108.

[132] M. Lu , M. Z. Hu , Sep. Purif. Technol. 2020, 236, 116312. 10.1016/j.seppur.2019.116312.

[133] Y. Jia , S. Maitra , V. Singh , Bioresour. Technol. 2023, 371, 128630. 10.1016/j.biortech.2023.128630. 36657588

[134] N. Mohamad , M. Reig , X. Vecino , K. Yong , J. L. Cortina , J. Chem. Technol. Biotechnol. 2019, 94 (9), 2899–2907. 10.1002/jctb.6093 (acccessed 2024/09/02).

[135] T. Wang , Y. Meng , Y. Qin , W. Feng , C. Wang , J. Energy Inst. 2018, 91(3), 473–480. 10.1016/j.joei.2017.01.005.

[136] H. Wu , T. Kim , S. Ferdous , T. Scheve , Y. Lin , L. Valentino , M. Holtzapple , T. R. Hawkins , P. T. Benavides , M. Urgun-Demirtas , ACS Sustain. Chem. Eng. 2024, 12(18), 6990–7000. DOI: 10.1021/acssuschemeng.4c00167.

[137] F. A. Saadi , M. I. Nelson , Proceedings of the First International Conference on Aeronautical Sciences, Engineering and Technology, Singapore, 2024//, (Eds: A. A. Khan , M. S. Hossain , M. Fotouhi , A. Steuwer , A. Khan , D. F. Kurtulus ), Springer Nature, Singapore 2024, 296–301.

[138] A. S. Dotsenko , Y. A. Denisenko , A. M. Rozhkova , I. N. Zorov , I. A. Shashkov , Bioresour. Technol. Rep. 2022, 17, 100887. 10.1016/j.biteb.2021.100887.

[139] X. Liang , Y. Guo , Bioresour. Technol. 2022, 362, 127805. 10.1016/j.biortech.2022.127805. 36007766

[140] C. Abels , C. Redepenning , A. Moll , T. Melin , M. Wessling , J. Membr. Sci. 2012, 405–406, 1–10. 10.1016/j.memsci.2011.12.020.

[141] P. Dolejš , V. Poštulka , Z. Sedláková , V. Jandová , J. Vejražka , E. Esposito , J. C. Jansen , P. Izák , Sep. Purif. Technol. 2014, 131, 108–116. 10.1016/j.seppur.2014.04.041.

[142] P. Wojnarova , J. Rusin , P. Basinas , M. Kostejn , J. Nemec , P. Stanovský , A. S. Kim , P. Izak , Sep. Purif. Technol. 2023, 326, 124783. 10.1016/j.seppur.2023.124783.

[143] E. Esposito , L. Dellamuzia , U. Moretti , A. Fuoco , L. Giorno , J. C. Jansen , Energy Environ. Sci. 2019, 12(1), 281–289.

[144] B. Van der Bruggen , M. Mänttäri , M. Nyström , Sep. Purif. Technol. 2008, 63(2), 251–263. 10.1016/j.seppur.2008.05.010.

[145] G. Rudolph , T. Virtanen , M. Ferrando , C. Güell , F. Lipnizki , M. Kallioinen , J. Membr. Sci. 2019, 588, 117221. 10.1016/j.memsci.2019.117221.

[146] A. Naeem , B. Saeed , H. AlMohamadi , M. Lee , M. A. Gilani , R. Nawaz , A. L. Khan , M. Yasin , Sep. Purif. Technol. 2024, 336, 126271. 10.1016/j.seppur.2024.126271.

[147] S. P. Nunes , P. Z. Culfaz-Emecen , G. Z. Ramon , T. Visser , G. H. Koops , W. Jin , M. Ulbricht , J. Membr. Sci. 2020, 598, 117761. 10.1016/j.memsci.2019.117761.

[148] G. T. Szekely , RSC Sustain. 2024, 2(4), 871–880.

[149] A. Figoli , T. Marino , S. Simone , E. Di Nicolò , X. M. Li , T. He , S. Tornaghi , E. Drioli , Green Chem. 2014, 16(9), 4034–4059.

[150] P. Yadav , N. Ismail , M. Essalhi , M. Tysklind , D. Athanassiadis , N. Tavajohi , J. Membr. Sci. 2021, 622, 118987. 10.1016/j.memsci.2020.118987.

[151] W. Wang , X. Fenjuan , J. Wanqin , X. Nanping , Progr. Chem. 2007, 19(10), 1592–1597.

[152] B. K. Nandi , R. Uppaluri , M. K. Purkait , Appl. Clay Sci. 2008, 42(1), 102–110. 10.1016/j.clay.2007.12.001.

[153] K. Guerra , J. Pellegrino , Sep. Sci. Technol. 2013, 48(1), 51–65. DOI: 10.1080/01496395.2012.690808.

[154] Research, G. V. Membrane Separation Technology Market Size, Share & Trends Analysis Report By Technology (Microfiltration, Ultrafiltration, Nanofiltration, Reverse Osmosis), By Application, By Region, And Segment Forecasts, 2022–2030; https://www.grandviewresearch.com/industry-analysis/membrane-separation-technology-market.

[155] E. Favre, *Front. Chem. Eng*. **2022**, *4*, Perspective.

[156] M. J. Talukder , A. S. Alshami , A. Tayyebi , N. Ismail , X. Yu , Sep. Purif. Rev. 2024, 53(2), 216–229. DOI: 10.1080/15422119.2023.2212295.

[157] D. Yu , X. Xiao , C. Shokoohi , Y. Wang , L. Sun , Z. Juan , M. J. Kipper , J. Tang , L. Huang , G. S. Han , et al. Adv. Funct. Mater. 2023, 33(9), 2211983. DOI: 10.1002/adfm.202211983 (acccessed 2024/08/17).

[158] T. Huang , Z. Su , K. Hou , J. Zeng , H. Zhou , L. Zhang , S. P. Nunes , Chem. Soc. Rev. 2023, 52(13), 4173–4207. 37184537 10.1039/d2cs00911k

[159] E. Drioli , A. I. Stankiewicz , F. Macedonio , J. Membr. Sci. 2011, 380(1), 1–8. 10.1016/j.memsci.2011.06.043.

[160] S. E. Demirel , J. Li , M. M. F. Hasan , Ind. Eng. Chem. Res. 2021, 60(19), 7197–7217. DOI: 10.1021/acs.iecr.0c05072.

[161] F. J. Keil , Process Intensification 2018, 34(2), 135–200. DOI: 10.1515/revce-2017-0085 (acccessed 2024–08-31).

[162] Y. S. Lin , Ind. Eng. Chem. Res. 2019, 58(15), 5787–5796. DOI: 10.1021/acs.iecr.8b04539.

[163] W. J. Koros , R. P. Lively , AIChE J. 2012, 58(9), 2624.

